# Diversity, structure, and distribution of bacterioplankton and diazotroph communities in the Bay of Bengal during the winter monsoon

**DOI:** 10.3389/fmicb.2022.987462

**Published:** 2022-11-30

**Authors:** Chao Wu, Dhiraj Dhondiram Narale, Zhengguo Cui, Xingzhou Wang, Haijiao Liu, Wenzhe Xu, Guicheng Zhang, Jun Sun

**Affiliations:** ^1^Key Laboratory of Sustainable Development of Marine Fisheries, Ministry of Agriculture and Rural Affairs, Yellow Sea Fisheries Research Institute, Chinese Academy of Fishery Sciences, Qingdao, China; ^2^Laboratory for Marine Fisheries Science and Food Production Processes, Pilot National Laboratory for Marine Science and Technology, Qingdao, China; ^3^Research Centre for Indian Ocean Ecosystem, Tianjin University of Science and Technology, Tianjin, China; ^4^Institute for Advanced Marine Research, China University of Geosciences, Guangzhou, China; ^5^State Key Laboratory of Biogeology and Environmental Geology, China University of Geosciences, Wuhan, China

**Keywords:** Bay of Bengal, diazotrophs, marine bacterioplankton, heterotrophic bacteria, high throughput sequencing

## Abstract

The Bay of Bengal (BoB) is conventionally believed to be a low productive, oligotrophic marine ecosystem, where the diazotroph communities presumed to play a vital role in adding “new” nitrogen through the nitrogen fixation process. However, the diazotroph communities in the oceanic region of the BoB are still poorly understood though it represents most of the seawater volume. The present study investigated a detailed account of the bacterioplankton community structure and distribution in the oceanic BoB during the winter monsoon using high throughput sequencing targeting the 16S rRNA and *nif*H genes. Our study observed diverse groups of bacterioplankton communities in the BoB including both cyanobacterial and non-cyanobacterial phylotypes. Cyanobacteria (*Prochlorococcus* spp. and *Synechococcus* spp.) and Proteobacteria (mainly α-, γ-, and δ-Proteobacteria) were the most abundant groups within the bacterial communities, possessing differential vertical distribution patterns. Cyanobacteria were more abundant in the surface waters, whereas Proteobacteria dominated the deeper layers (75 m). However, within the diazotroph communities, Proteobacteria (mainly γ-Proteobacteria) were the most dominant groups than Cyanobacteria. Function prediction based on PICRUSt revealed that nitrogen fixation might more active to add fixed nitrogen in the surface waters, while nitrogen removal pathways (denitrification and anammox) might stronger in deeper layers. Canonical correspondence analysis (CCA) indicated that temperature, salinity, and silicate were major environmental factors driving the distribution of bacterial communities. Additionally, phosphate was also an important factor in regulating the diazotroph communities in the surface water. Overall, this study provided detailed information on bacterial communities and their vital role in the nitrogen cycles in oligotrophic ecosystems.

## Introduction

Seasonal reversal monsoon systems and the resulting meso- or basin-scale sea surface circulation patterns in the Bay of Bengal (BoB) are the most fascinating climatological features of the seas around the world (Schott and Mccreary, 2001; Vecchi and Harrison, 2002; Chen et al., 2018). The hydrography and circulation are influenced by the seasonal reversal monsoons in the BoB, with poleward flowing East Indian Coastal Current (EICC), eastward Southwest Monsoon Current during June-September (summer monsoon) and equatorward EICC, and westward Northeast Monsson Currents during December-February (winter monsoon) (Shankar et al., 2002; Miyama et al., 2003; Schott et al., 2009). This reversal pattern of monsoon currents plays a significant role in the water mass exchange between the BoB and the Arabian Sea (AS) (Shankar et al., 2002; Prasanna et al., 2004). Except for the two monsoons, winds over the northern Indian Ocean are generally weak and consistent westerly during the transition (inter) monsoon periods, i.e., spring (March-May) and fall (September-October) (Schott et al., 2009). The surface water during these inter-monsoon periods is weakly churned by the persistently steady zephyr, and hence most regions in the bay are calm, stratified, and oligotrophic (Narvekar and Prasanna, 2006, 2014; Jinadasa et al., 2020). In general, winds in the northern Indian Ocean are strongest in the summer monsoon, intense in the winter monsoon, and weakest in the transition periods (Vecchi and Harrison, 2002; Singh and Ramesh, 2015).

Though the BoB is located in the same latitudinal belt as the AS, the former is influenced by a more intense monsoon rainfall and river runoff (Prasanna et al., 2002; Wijesekera et al., 2015; Sarma et al., 2016). The immense annual freshwater runoff from the Himalayan Rivers (Ganges and Brahmaputra) and other peninsular rivers (Mahanadi, Krishna-Godavari, etc.) into the BoB decreases average surface salinity on a large scale (Prasanna et al., 2002; Narvekar and Prasanna, 2014; Jinadasa et al., 2020). The freshwater capping creates a strong and highly stable “barrier layer” in the upper layers of the northern BoB, thereby inhibiting the vertical turbulent transport of nutrients, heat, momentum, and tracers (Prasanna et al., 2002; Kumari et al., 2018). Even under strong summer monsoon wind forcing, surface water in the BoB is weakly churned, and hence restricts the vertical transport of nutrients. More than that, major physical processes such as summer coastal upwelling and winter convective mixing are very weak in the BoB, but alternatively, eddies, gyres, and other episodic events dominate in this region and make the bay locally productive (Shetye et al., 1991; Prasanna et al., 2002, 2004; Vidya and Kumar, 2013; Singh et al., 2015; Chen et al., 2018). In brief, the BoB is conventionally believed to be an oligotrophic system relative to the AS, partly due to the depletion of surface nutrients, intense cloud cover, narrow shelf region, and high turbidity waterbody jointly restricting the growth of phytoplankton (Prasanna et al., 2002; Madhupratap et al., 2003; Gauns et al., 2005; Narvekar and Prasanna, 2014; Bhushan et al., 2018).

The other characteristic of the BoB is that subsurface waters contain extremely low but persistent concentrations of dissolved oxygen (DO) (Löscher et al., 2020). Low DO concentrations have profound influences on the marine microbial community which could eventually change marine biogeochemical cycling such as nitrogen and sulfur cycling (Cheung et al., 2015; Fernandes et al., 2020). Bristow et al. (2016) found that OMZs in the BoB support denitrifies and anammox microbial communities, mediating low but significant nitrogen loss. One more recent research reported a significant variation in nitrogen and sulfur metabolism in the OMZs of the BoB (Fernandes et al., 2019, 2020). Further analysis demonstrated that δ-Proteobacteria and γ-Proteobacteria affiliated with Proteobacteria showed high abundance in the deoxygenated waters in OMZs of BoB (Fernandes et al., 2020). Bacterial communities have also been explored in the coastal and offshore waters of the BoB, with *Synechococcus*, *Erythrobacter*, and *Psychrobacter* dominating in the coastal region while *Prochlorococcus* and *Vibrio* in the offshore waters (Vijayan et al., 2020). However, these studies focused on specific regions (e.g., coastal region, offshore region, and OMZs), no study was conducted in the open regions though they represent most of the seawater volume of the BoB.

Most parts of the BoB, especially in the open region, are lacking bioavailable nitrogen in the surface waters (Löscher et al., 2020). Thus, the nitrogen-transforming microorganisms which control the balance of bioavailable nitrogen are particularly important in this oligotrophic ecosystem (Wu et al., 2018, 2019). Diazotroph communities play a vital role in sustaining primary productivity by adding new nitrogen to oligotrophic marine ecosystems (Levitan et al., 2007; Gradoville et al., 2017). Earlier studies had reported that the globally significant marine nitrogen fixer *Trichodesmium* spp. inhabits the northern Indian Ocean throughout the year (Hegde et al., 2008; Achary et al., 2014; Sahu et al., 2017). Particularly, this genus forms periodic blooms in the BoB during the spring inter-monsoon periods (Li et al., 2012; Jyothibabu et al., 2017). Compared with the high frequency of blooms during the inter-monsoon season, *Trichodesmium* spp. blooms are a small probability event in the monsoon seasons. Many studies showed that *Trichodesmium* spp. usually thrives in a relatively stable environment (Jyothibabu et al., 2017; Wu et al., 2018). The unusually strong winds and high turbulent surface water during the active monsoon seasons are not favorable for the flourishing of *Trichodesmium* spp. In addition to *Trichodesmium* spp., symbiotic diatom-diazotrophic cyanobacteria associations were also reported widely distributed in the northern Indian Ocean (Madhu et al., 2013; Manjumol et al., 2018). Other diazotrophs, for instance, unicellular diazotrophic cyanobacteria (UCYN) and heterotrophic diazotrophs (various Proteobacteria populations), are less studied in the BoB.

This study presents a detailed account of bacterioplankton communities (including diazotrophs) in the oligotrophic BoB waters during the winter monsoon. Furthermore, to better understand the variation within the diazotrophs in different months, the result was compared with the earlier study carried out during the spring inter-monsoon (Wu et al., 2019). The high throughput sequencing was used to assess the detailed information of dominant, rare species and many uncultured bacterioplankton populations. In addition, quantitative methods including real-time fluorescent quantitative polymerase chain reaction (qPCR) assay and microscope counting were also used to determine the factual abundance of diazotrophs. Thus, overall, this study will enhance our understanding of spatial-temporal heterogeneity and distribution of bacterioplankton communities and their vital roles in nitrogen cycling in oligotrophic marine ecosystems.

## Materials and methods

### Sampling strategies

**FIGURE 1:**
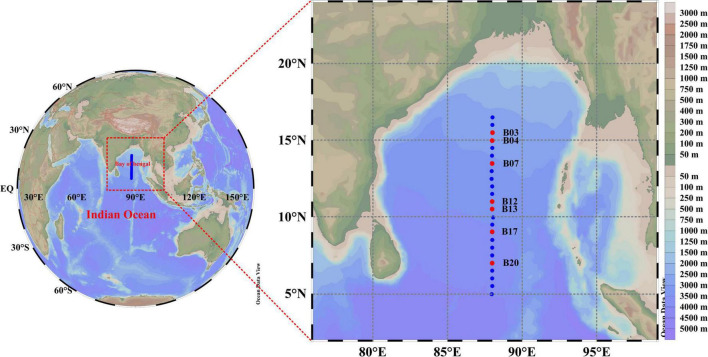
The map showing the sampling stations in the central BoB. The left miniature map describes the location of the BoB. The blue dots in the right map showing stations used for hydrography measurement, among which red dots represent stations used for the molecular experiment.

To better understand the bacterioplankton communities in the BoB, sampling was carried out during R/V “*Dongfanghong II*” (8 October to 20 December 2016) at 24 stations located between 2 and 18°N along 88°E (Figure 1). All 24 stations were sampled for the assessment of the environmental parameters, whereas molecular samples were collected only at seven selected stations (details in Figure 1). Water samples were collected at 6 depths (0, 30, 75, 100, and 150–300 m) using 12-L Go-Flo bottles attached to a rosette multi-sampler installed with CTD probes (Seabird SBE 911Plus, Sea-Bird Electronics, Inc., Bellevue, WA, USA). Environmental parameters such as temperature and salinity were recorded vertically with the CTD sensor. The vertical profiles of the environmental parameters were visualized by Ocean Data View (ODV V5.0.0; Schlitzer, 2008). Collected water samples were transferred into 15-L HCl-rinsed buckets. For nutrient measurement, subsamples were transferred into 100-ml HCl-rinsed bottles and kept in a refrigerator until further processing. To quantify the filamentous cyanobacteria, a 1-L water sample was fixed with 1% formaldehyde in a plastic bottle and stored in dark. For chlorophyll a (Chl*a*) analysis, 1-L subsamples were vacuum-filtered (<100 mm Hg) through a 25-mm Whatman™ GF/F filter (Whatman, Florham Park, NJ, USA). The filters were packed in aluminum foil to prevent light and stored at 4°C until further analysis. For molecular analysis, 2–4 L subsamples were filtered through 0.22-μm polycarbonate filters (Millipore, Eschbonn, Germany) under a low-pressure vacuum (<100 mm Hg). The filters were placed into 20-ml microtubes, flash-frozen immediately in liquid nitrogen, and stored at –80°C in the lab until analysis.

### Environmental parameter measurement

The nutrients, including nitrate (${\text{NO}}_{3}^{-}$), nitrite (${\text{NO}}_{2}^{-}$), ammonium (NH_4_), and phosphate (${\text{PO}}_{4}^{3-}$), were analyzed with Technicon AA3 Auto-Analyzer (Bran+ Luebbe) using the standard methods (Grasshoff et al., 1982). ${\text{NO}}_{3}^{-}$, ${\text{NO}}_{2}^{-}$, and NH_4_ were measured using the copper-cadmium column reduction methods, while ${\text{PO}}_{4}^{3-}$ was measured by typical spectrophotometric methods. For Chl*a* estimation, filters were extracted using the acetone (90%) extraction method. The Chl*a* concentrations were measured using a Trilogy (CHL NA, Model # 046) fluorometer.

### Deoxyribonucleic acid extraction, polymerase chain reaction amplification, and sequencing

Genomic DNA was extracted using DNeasy PowerWater^®^ Kit (Qiagen, Hilden, Germany) following the manufacturer’s protocol. The quality and quantity of the extracted DNA were measured using an ND-2000 Nanodrop spectrometer (Thermal Scientific, Wilmington, DE, USA). A total of 14 samples from two depths (0 and 75 m) at each station were used for high throughput sequencing analysis. Two genes (16S rRNA and *nif*H) were amplified from the 14 samples in the present study. To characterize the total bacterioplankton composition, the V3–V4 region of the 16S rRNA gene was amplified with the pair-wise common primer 338F (5′-ACTCCTACGGGAGGCAGCA-3′) and 806R (5′-GGACTACHVGGGTWTCTAAT-3′) (Mori et al., 2014). Reactions contained 5X Q5 Reaction Buffer (10 μl), 5X Q5 High GC Enhancer (10 μl), 10 μM forward and reverse primers (1.5 μl each), 10 μM dNTPs (1 μl), Q5 High-Fidelity DNA Polymerase (0.2 μl) (New England Biolabs, Whitby, ON, Canada), 40–60 ng of DNA templates, and nuclease-free water to adjust the final volume of 50 μl. The thermal profile used for the 16S rRNA gene amplification was denatured (95°C, 5 min), followed by 25 cycles of denaturation (95°C, 30 S), annealing (50°C, 30 S), and extension (72°C, 40 S) with a final extension (72°C, 7 min). The diazotroph community composition was defined by the *nif*H gene. The *nif*H gene fragment was amplified by nested PCR using the protocol given by Zehr et al. (1998). The details of the PCRs reaction system and thermal profile are illustrated by Wu et al. (2019). Note that negative controls were set up by replacing template DNA with nuclease-free water. All the PCR amplifications were conducted in a Veriti 9902 thermocycler (Applied Biosystems, Foster City, CA, USA). Remarkably, primers used in the 16S rRNA gene amplification and the second round of the *nif*H gene amplification were composed of dual-indexed barcodes to distinguish different samples. After amplification, all the PCR products were checked in 1.8% agarose gel. Samples with bright bands of approximately 450 bp for the 16S rRNA gene and 360 bp for the *nif*H gene were thought to be successfully amplificated. In addition, negative controls have no obvious bands that were considered contamination free. The PCR products were purified by MinElute^®^ PCR Purification Kit (Qiagen, Hilden, Germany), and the purified PCR products were validated using Nanodrop 2000. Finally, the libraries were sequenced using paired-end chemistry (PE250) on an Illumina Hiseq2500 platform (Illumina, San Diego, California, USA) at Biomarker Technologies, Beijing, China. The 16S rRNA gene and *nif*H gene raw sequences obtained from this study were deposited in NCBI Sequence Read Archive with BioProject no. PRJNA637963 and PRJNA637983, respectively.

### Quality control and bioinformatics analysis

The 16S rRNA gene data were analyzed in the BMK Cloud.^1^ The detailed procedure of the bioinformatics analysis for the 16S rRNA gene is given in Wu et al. (2022a). For *nif*H gene analysis, the raw sequence data were first quality filtered to remove low-quality tags. Then these sequences were separated by samples, according to their barcode sequences, permitting up to one mismatch (Zhang et al., 2017). The paired-end reads were subsequently merged into full-length sequences by FLASH v1.2.7 software to get raw tags (Magoč and Salzberg, 2010). The minimal overlapping length and maximum mismatch ratio in this step were 10 bp and 0.2, respectively. Notably, the paired-end reads without overlaps were removed from the pool. To remove low-quality raw tags, Trimmomatic v0.33 software was used to filter sequences less than 300 bp in length, or that contained homopolymers longer than 8 bp and ambiguous base-pair (Huse et al., 2007; Kunin et al., 2010; Bolger et al., 2014). The chimera sequences were also removed from the raw tags by comparing tags with the reference database in the UCHIME v4.2 software (Edgar et al., 2011), and the remaining effective tags were grouped into operational taxonomic units (OTUs) at 97% similarly by USEARCH v10.0 (Edgar, 2010). In the present study, the most common sequences in each OTU were selected as representative sequences.

For taxonomic classification of the diazotroph communities, representative sequences were first translated into amino acid sequences and searched in the protein sequences database of the National Center for Biotechnology Information (NCBI) using BLASTX v2.8.1+ (Altschul et al., 1997). The most closely related sequences (>96% similarly) were chosen as the alignment sequences. Finally, the representative and alignment sequences were aligned with ClustalW in MEGA v7.0, and a phylogenetic neighbor-joining tree was subsequently constructed using the maximum likelihood method (Kumar et al., 2016). Bootstrap values were determined by resampling 1,000 times, and bootstrap values greater than 50% were shown near nodes. The constructed tree was further edited by the Interactive Tree of Life (iTOL), an online tool for managing the phylogenetic tree (Letunic and Bork, 2011).

### Statistical analysis

Rarefaction curves were calculated by Past V3.0 software and further visualized in the online software based on the standard operating procedure shown on the website.^2^ Alpha and beta diversity were calculated based on the OTU tables for comparing the relative complexity of bacterioplankton communities. Alpha diversity including the Chao1 richness estimator, Shannon-Weiner diversity index, and Simpson similarity index were calculated in the R V3.3.2 software. Non-metric multidimensional scaling (NMDS) analysis was conducted in PRIMER V6.0 software to characterize the vertical and horizontal distribution patterns of bacterioplankton communities (Clarke and Gorley, 2006). To evaluate the relationship between environmental factors and bacterial-diazotroph communities, canonical correspondence analysis (CCA) was carried out using CANCO 4.5 software. The detailed procedure of CCA is given by Kong et al. (2011) and Wu et al. (2019). The network was used to explore the co-occurrence patterns of bacterioplankton communities by Gephi v0.9.2. For convenience, only the OTUs with relative abundance greater than 0.1% were selected for network analysis (Jiao et al., 2016). The possible pairwise Spearman’s rank correlation (*r*) between OTUs was calculated within the package of *psych* in R V3.3.2 software. Only statistically robust (|*r*| > 0.9 for bacterial communities, and | *r*| > 0.7 for diazotroph communities) and significant (*P* < 0.01) correlations were included in the further analysis. The topology of the network correlations was visualized in Gephi. Furthermore, node-level topological properties were also obtained in the Gephi. Nodes with high degree and low betweenness centrality values in the networks were identified as keystone species (Wu et al., 2022a). The functions of bacterial communities were predicted by PICRUSt genome prediction software v0.9.2 (Natale et al., 2000). The predicted proteins related to nitrogen cycles were discovered and visualized by heatmap using R v3.6.1 software.

### Quantification of *nif*H phylotypes

The filamentous cyanobacteria population was microscopically quantified and enumerated from the preserved seawater samples. Prior to the analysis, the 1-L seawater sample was concentrated to a 200 ml solution by siphoning method (Liu et al., 2020). Then, 100-ml concentrated solution was transferred into an Utermöhl chamber for 24 h sedimentation (Uthermöhl, 1958). Finally, the filamentous cyanobacteria and their corresponding cells were identified and counted under an inverted microscope (Motic, AE2000) using 200 or 400 magnifications.

The quantity of unicellular diazotrophs, qPCR targeting UCYN-A, UCYN-B, and two uncultured proteobacteria were conducted using an ABI Step One Plus Real-Time PCR System (Applied Biosystems, Foster City, CA, USA). The corresponding *nif*H standards were obtained from the clone library of environmental samples, except for the alpha-proteobacteria, which directly use the specific primers amplified from the environmental samples. The specific primers, probes, and standard clones used in the present study are described in Table 1. The qPCR reactions were performed in duplicate in a final volume of 10 μl which contained 5 μl 2 × Premix Ex Taq™ (Takara Bio, Tokyo, Japan), 50 × ROX Reference Dye (0.2 μl), 10 μM forward and reverse primers (0.4 μl), TaqMan probe (0.4 μl), template DNA (1 μl), and finally nuclease-free water to adjust the final volume. qPCR condition was denatured (95°C, 30 S), followed by 45 cycles of denaturation (95°C, 5 S), and annealing (60°C, 30 S) (Wu et al., 2019). Standard curves were determined by 10-fold dilution series from 10 to 10^7^ gene copies per reaction. The linear regression (*R*^2^) values and amplification efficiencies (*E*) of each standard curve greater than 0.99 and 90% were thought effective. The amplification efficiency was calculated by the equation *E* = 10^–1/^*^m^* – 1, where m is the slope of the standard curve. Non-target templates were also tested in the same condition as the standards and samples. Where amplification of non-target templates occurred (Ct values ranged from 35 to 38), the non-target template gene copies were subtracted from the sample values to adjust for slight contamination. The depth-integrated gene abundances were computed by trapezoidal integration over the sampling depth.

**TABLE 1:** Primers, Taqman probes, and standard clones for quantitative polymerase chain reaction (qPCR) analysis targeting the *nif*H gene of different cyanobacterial diazotrophic groups.

Targets	Forward primer (5′–3′)	Probe	Reverse primer (5′–3′)	References
UCYN-A	GGTTACAACAACGTTTTATGTGTTGA	TCTGGTGGTCCTGAGCCCGGA	GCAGTAATAATACCACGACCAGCAC	Goebel et al. (2010)
UCYN-B	TGGTCCTGAGCCTGGAGTTG	TGTGCTGGTCGTGGTAT	CTTCTTCTAGGAAGTTGATGGAGGTG	Goebel et al. (2010)
*Sagittula castanea*	ATCACCGCCATCAACTTCCT	CGCCTACGATGACGTGGATTACGTGTCC	AGACCACGTCGCCCAGAAC	Zhang et al. (2011)
γ-HM210363	CCTGGACTTCGTATTCTA	CGATGTTGTATGCGGTGGCTT	CCACAATGTAGATTTCCTG	Halm et al. (2012)

The 5′ and 3′ of TaqMan probes were labeled with the fluorescent reporter FAM (6-carboxyfluorescein) and the quenching dye TAMRA (6-carboxytetramethylrhodamine), respectively.

## Results

### Hydrography and environmental parameters

**FIGURE 2:**
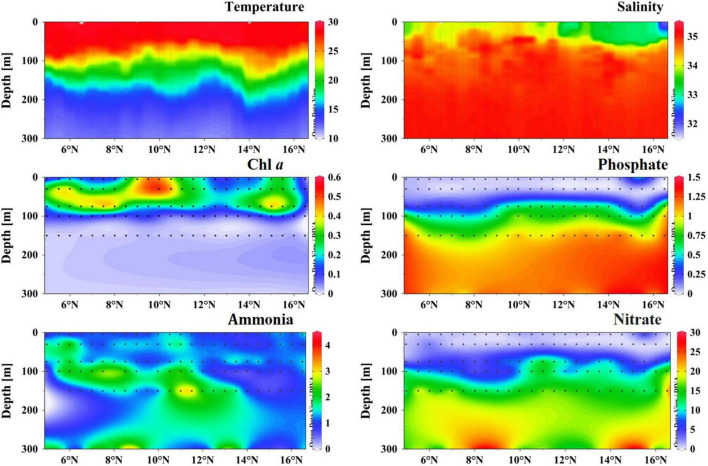
Vertical profiles of temperature (°C), salinity, Chl*a* (μg/L), phosphate (μM), ammonia (μM), and nitrate (μM) concentration in the sampling transect.

Hydrography and environmental parameters were supplied in Figure 2 and Supplementary Table 1. During the study period, the sea surface temperature (SST) ranged from 27.7 to 29.3°C with an average of 28.7°C and showed an equatorward increasing trend (Figure 2). The thermocline was estimated between 75 and 125 m across this transect, whereas temperature decreased drastically under the thermocline. Overall, sea surface salinity (SSS) ranged from 31.754 to 34.312 (average 33.642) and revealed significant regional variation (Figure 2). Two distinct low SSS areas were observed at both ends of the transect (5 and 16°N), partly due to the freshwater runoff. The halocline was limited between 75 and 100 m, and the halocline depth increased toward the equator (Figure 2). The Chl*a* concentration in the surface water varied between 0.03 μg/L (at Sta. B01) and 0.53 μg/L (Sta. B15). Chl*a* maximum layer was estimated at approximately 50 m except for 10°N, where a typical high value was observed in the upper 50 m (Figure 2). Furthermore, Chl*a* concentration was almost undetectable less than 130 m. The surface nutrient concentrations, i.e., phosphate (0.01–0.574 μM), ammonia (0.629–1.571 μM), and nitrate (0.294–2.070 μM) were comparatively low, which indicated the BoB as a typical oligotrophic ecosystem (Figure 2).

### Bacterioplankton community structure and diversity using 16S rRNA gene

In total, 429,576 high-quality sequences were obtained from the 16S rRNA gene sequencing, which clustered into 573 OTUs at 97% sequence similarity. According to the rarefaction curves, we observed that most samples were plateaued (Supplementary Figure 1). The alpha diversity of the 16S rRNA gene was shown in Supplementary Table 2. Good’s coverage of all 16S rRNA gene sequencing was greater than 99.8%, revealing full rarefaction saturation of all the samples and recovery of most bacterioplankton taxa in samples. The OTU numbers ranged from 299 to 353 in 0 m and 417–470 in 75 m samples. In general, the surface samples presented to low OTUs than the 75 m samples (one-way ANOVA, *P* < 0.01). Shannon-Weiner and Simpson diversity indices also revealed that the 75 m samples owned higher species abundance than the surface samples (one-way ANOVA, *P* < 0.01).

**FIGURE 3:**
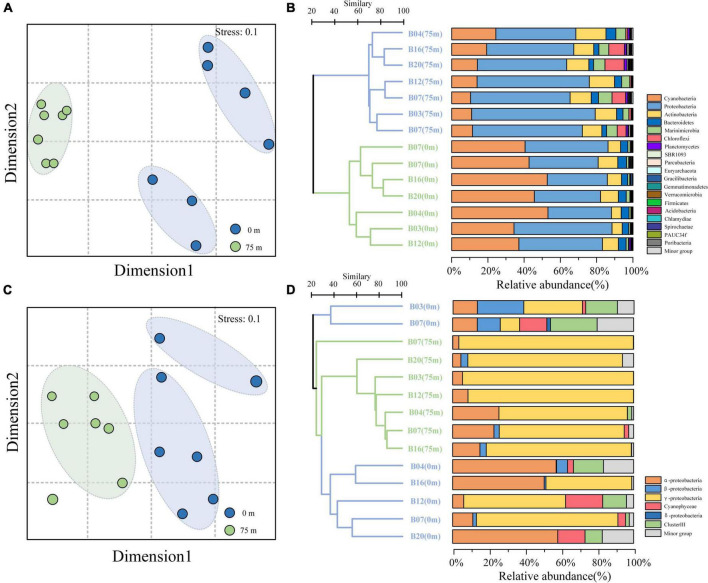
Non-metric multidimensional scaling (NMDS) analysis and cluster analysis of the bacterial **(A,B)** and diazotroph community composition **(C,D)** in the BoB. Samples from different depths were indicated by color.

Non-metric multidimensional scaling (NMDS) analysis revealed an obvious separation of the 16S rRNA gene in the surface and deeper layer (Figure 3A). The predominated phylum in the study area was Proteobacteria (48.8% of the total 16S rRNA gene sequence), followed by Cyanobacteria (29.8%), Actinobacteria (10.1%), Bacteriodetes (3.9%), and Marinimicrobia (3.2%) (Figure 3B). These top five phyla accounted for more than 95.8% of the total bacterial communities across all the samples in the BoB. Other bacterial communities, such as Chloroflexi and Planctomycetes, were also detected but in very low relative abundance. Proteobacteria was the most diverse and abundant bacterial phylum across all the samples (53.6% of OTUs). Among which, the α-Proteobacteria (20.8%), γ-Proteobacteria (17.6%), and δ-Proteobacteria (13.6%) were the most diverse Proteobacteria in the BoB, accounting for 52% of the total number of OTUs. The relative abundance of Proteobacteria contributed about 48.8% to the total sequences and showed little difference between the two depths (22.2% on the surface and 26.6% in the 75 m layers). The α-proteobacteria was the most abundant Proteobacteria at each station (27% of the total sequences) and revealed a little difference in relative abundance among the two depths. However, γ-Proteobacteria and δ-proteobacteria were distinct from the α-proteobacteria, with more abundant in the deep layer. Unlike Proteobacteria, Cyanobacteria were more abundant in the surface water (22.7 and 7.1% of the total sequence in 0 and 75 m, respectively). Among the Cyanobacteria, *Prochlorococcus* always dominated the surface layers, whereas *Synechococcus* showed minor differences within the two depths. The dominant subgroup of the Actinobacteria, *Candidatus*, and *Actinomarina* was also detected in all samples and accounted for 5.4% of the total sequences. The rest subgroups of Actinobacteria have occurred in extremely low abundance. Flavobacteria represented the most fractions of Bacteriodetes, which accounted for 3.8% of the total sequence. Marinimicrobia was another dominant group in the bacterial communities, represented solely by the clade SAR406.

### Diazotroph community structure and diversity using *nif*H gene

The rarefaction curves of the *nif*H gene were supplied in Supplementary Figure 1. We found that many OTUs in two samples (B07, 75 m and B20, 75 m) only contained 2 sequences from the OTU table. Thus, here it can be hypothesized that some errors might occur in the process of sequencing. To decrease the influence of these errors, OTUs that contained less than 10 sequences across all samples were removed from the OTU table. The alpha diversity of the *nif*H gene is given in Supplementary Table 2. In total, 901,603 high-quality sequences and 495 OTUs were included in our analysis. The sequencing coverages (C) were all greater than 99%, which suggested that the sequencing was deep enough for *nif*H gene diversity analysis in the present study. Unlike with 16S rRNA sequencing, the *nif*H gene OTUs in the surface samples were greater than the 75 m samples (one-way ANOVA, *P* < 0.05). Shannon-Weiner and Simpson diversity indices also showed the same vertical variation pattern (one-way ANOVA, *P* < 0.05).

**FIGURE 4:**
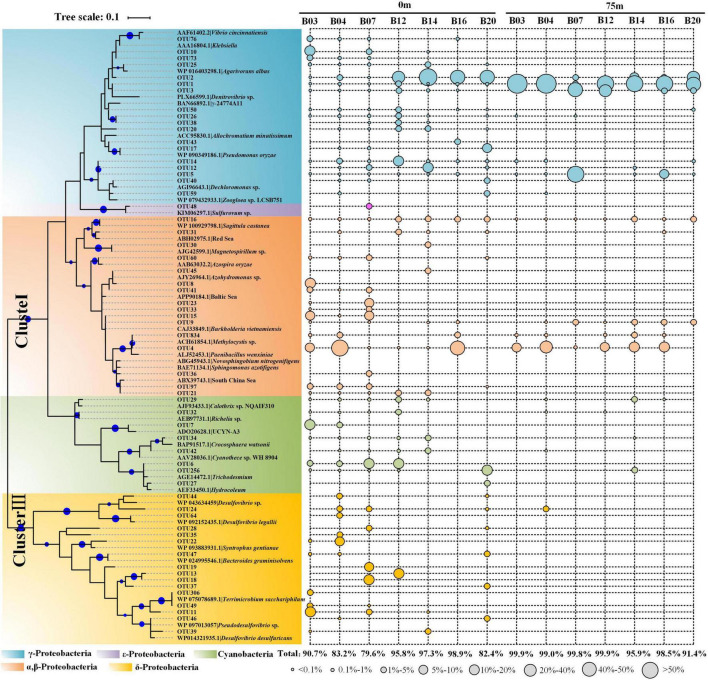
Neighbor-joining phylogenetic tree constructed by top 59 *nif*H gene amino acid sequences obtained from the BoB and reference sequences selected from the protein sequences database in GenBank. The topology of the tree was inferred from 1,000 bootstrap resampling, and bootstrap values greater than 50% were labeled with blue circle symbols on the branches. The circle symbols with different sizes represented the relative abundance of each OTU.

As the 16S rRNA gene, the *nif*H gene also presented obvious separation in the surface and deeper layer (Figure 3C). For the taxonomic classification of the OTUs, a neighbor-joining phylogenetic tree was constructed (Figure 4). The OTUs containing sequences greater than 1,000 were defined as top OTUs. The top 59 OTUs, which accounted for more than 94.4% of the total sequences, were included in the present analysis. In addition, 37 representative sequences were also selected from GenBank for comparison. Here, Figure 3D together with Figure 4 revealed the predominance of *γ-Proteobacteria* (63.4% of the total *nif*H gene sequence) in the *nif*H phylotypes in the BoB, followed by α-Proteobacteria (16.7%), δ-Proteobacteria (5.9%), Cyanophyceae (4.4%), and β-Proteobacteria (3.7%). γ-Proteobacteria was more abundant in the 75 m layer than the surface (one-way ANOVA, *P* < 0.01). On the contrary, *Cyanophyceae* and ClusterIII species were more abundant on the surface than the 75 m layer (one-way ANOVA, *P* < 0.05). However, no significant difference was observed in the occurrence of α-Proteobacteria within the two layers. From the phylogenetic tree (Figure 4), we observed that γ-Proteobacteria was the most diverse diazotroph communities (18 / 59 OTUs). OTU1 was the most abundant γ-Proteobacteria in the 14 samples. OTU1 showed 100% similarity to HM210363, a diazotroph reported from the south pacific gyre (Halm et al., 2012). OTU50 showed 100% identity with γ-24774A11 which was once reported as dominant in the AS during winter monsoon (BAN66892). However, in the present study, OTU50 was only detected in the surface waters at stations B12 and B20. In addition, many unidentified γ-Proteobacteria were also presented in high abundance. Here, α-Proteobacteria such as OTU4 and OTU16 were the most prevalent in the BoB. OTU4 had 100% identity with the isolation of *Methylocystis* strain m261 (ACH61854) from freshwater. OTU16 had 100% similarity with an uncultured α-Proteobacteria clone CE1-100m-2 (HQ586738) detected in the South China Sea, and 99% similarity with new isolation belonged to the genus of *Sagittula* from an OMZs in the eastern tropical South Pacific (CP021913). The dominance of *Sagittula* sp. was reported in the Eastern Indian Ocean (EIO) earlier during the spring inter-monsoon. In the BoB, however, OTU16 was not the dominant species. The neighbor-joining phylogenetic tree illustrated that 16 OTUs belonged to ClusterIII among the top 59 OTUs, including diverse *δ-*Proteobacteria. The *δ-Proteobacteria* was mainly observed in the surface waters and detected very rarely in the 75 m layers (Figure 4). Together with diverse *Proteobacteria*, we also identified 7 OTUs belonged to *Cyanophyceae*, including OTU6 (*Trichodesmium* sp.), OTU7 (UCYN-A3), OTU29 (*Calothrix* sp.), OTU34 (*Crocosphaera* sp.), OTU32 (*Richelia* sp.), OTU256, and OTU27. As expected, *Cyanophyceae* was not prevalent in the BoB (4.4% of the total sequences) during the winter monsoon. Among the 7 OTUs, *Trichodesmium* sp. and UCYN-A3 were the most abundant *Cyanophyceae* in the study region, but the two *Cyanophyceae* were only detected in the surface layer.

### Co-occurrence pattern of bacterial and diazotroph communities

**FIGURE 5:**
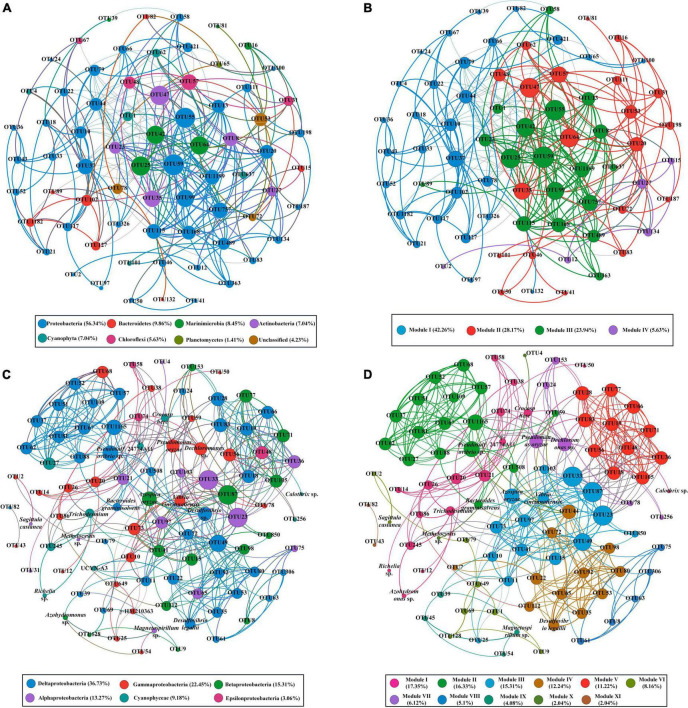
The co-occurrence patterns of bacterial communities **(A,B)** and diazotroph communities **(C,D)** revealed by network analysis. Only statistically robust (|*r*| > 0.9 for bacterial communities and |*r*| > 0.7 for diazotroph communities) and significant (*P*-value < 0.01) correlations were included in the further analysis. The nodes were colored according to different types of phylum **(A,C)** and modules **(B,D)**. The size of each node is proportional to the degree of nodes.

To understand the organization of the bacterial and diazotroph communities, network analysis was conducted based on the high-throughput sequencing data (Figure 5). Detailed information on node-level topological properties is presented in Table 2. The bacterial community network was composed of 71 nodes and 289 edges with a mean of 8.141 edges per node (Table 2). The average path length (APL) was 2.816 edges with a diameter of 8 edges. The modularity index was greater than 0.4 which implied the network for bacterial has a modular structure. In brief, our results indicated that the bacterial communities were composed of highly connected genera and formed distinguishable modules. The network was composed of six modules, in which *Proteobacteria* was the most abundant Phylum (Figure 5A). The further modularized analysis revealed that the network was mainly composed of four modules (Figure 5B). The nodes from module I was mostly composed of Proteobacteria and Bacterodetes; nodes from module III mostly belonged to Proteobacteria and Marinimicrobia (Figures 5A,B). Based on the centrality scores, the top five OTUs were identified as keystone taxa including OTU47 (Actinobacteria), OTU25 (Marinimicrobia), OTU64 (Marinimicrobia), OTU78 (Unclassified), and OTU102 (Bacteroidetes). For diazotroph communities, the network was composed of 98 nodes and 411 edges (Table 2). The average edges for each node were 10.299. Similar to the bacterial network, the modularity index was also greater than 0.4 which implies a good modular structure for diazotroph communities. The APL of the diazotroph network was 3.447 edges with a diameter of 9 edges. From the network, nodes were assigned to six classes (Figure 5C). Among these, δ-Proteobacteria accounted for about 36.73% of all nodes and presented high-level connectedness, indicating the vital ecological niche of these low abundance species. According to the value of betweenness centrality, six nodes were recognized as keystone species, including OTU49 (δ-Proteobacteria), OTU15 (β-Proteobacteria), OTU21 (α-Proteobacteria), OTU20 (γ-Proteobacteria), OTU74 (ε-Proteobacteria), and OTU60 (δ-Proteobacteria). All keystone species belonged to Proteobacteria in the present study. After modularizing, these nodes were grouped into 11 major modules (Figure 5D). We found that most of the cyanobacteria were grouped into Module I, and most of the *δ-Proteobacteria* were grouped into Module II (Figures 5C,D).

**TABLE 2:** Topological properties of the co-occurring networks of bacterial and diazotroph communities.

	Nodes	Edges	MD	CC	APL	ND	AD	GD
Bacterial network	71	289	0.808	0.528	2.816	8	8.141	0.116
Diazotroph network	98	411	0.700	0.670	3.447	9	8.388	0.086

MD, modularity; CC, clustering coefficient; APL, average path length; ND, network diameter; AD, average degree; GD, graph density.

### Nitrogen cycles simulation in the Bay of Bengal using PICRUSt

**FIGURE 6:**
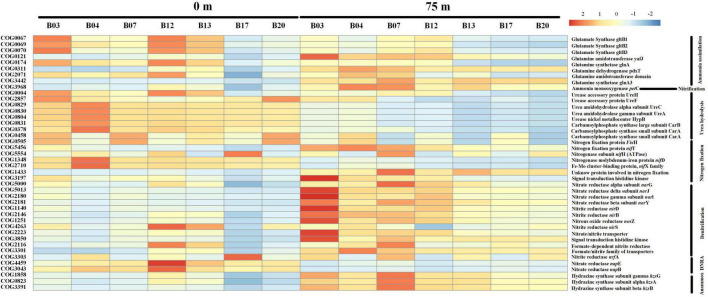
Heat map showing the relative abundance of different nitrogen metabolic enzymes and their corresponding genes based on the COG database. Notably, the relative abundance of the predicted proteins was log-transformed in the heatmap.

The proteins related to nitrogen cycles were predicted and visualized in Figure 6. According to the COG orthologs, the denitrification and ammonium oxidation (anammox) in the 75 m layers were decreased from north to south (Sta. B03 to Sta. B20), which implies the BoB losing fixed nitrogen in deeper layers. However, the two nitrogen-removed pathways were not active in the surface water. Ammonia oxidation, the first step of nitrification, was also detected by PICRUSt. It was also presented as more active in the 75 m layers than that in the surface water. Dissimilatory nitrate reduction to ammonium (DNRA) and urea hydrolysis were also detected in this study and were only active in the surface waters. Unlike denitrification and anammox, nitrogen fixation was more active in the surface waters. However, nitrogen fixation was also detected in the deeper layer and replenished some of the nitrogen loss.

### Quantification of cyanobacterial *nif*H phylotypes

**FIGURE 7:**
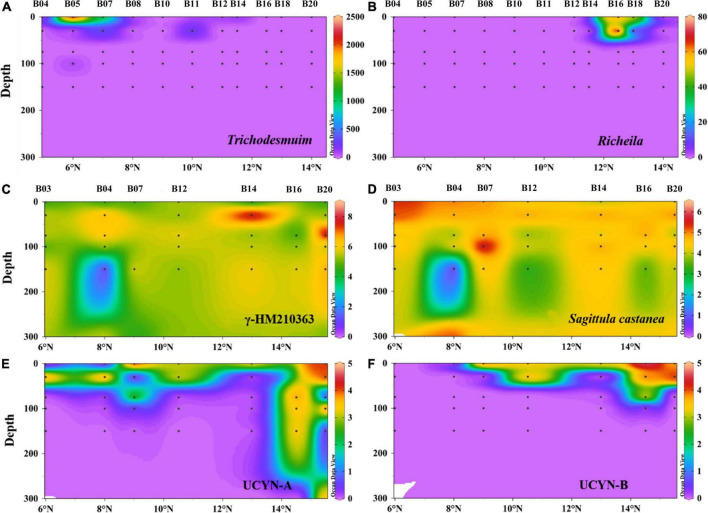
Depth profiles of filamentous cyanobacteria abundance (cell L^–1^) for *Trichodesmium* **(A)** and *Richelia intracellularis* **(B)** and *nif*H gene abundance (log_10_ copies L^–1^) for γ-HM210363 **(C)**, *Sagittula castanea* **(D)**, UCYN-A **(E)**, UCYN-B **(F)** in the BoB revealed by qPCR. For convenience, one gene copy used to represent where *nif*H genes were under detection.

Depth profiles of six main diazotrophic phylotypes are shown in Figure 7. *Trichodesmium* and *Richelia intracellularis* were only detected in the upper layer from 0 to 30 m, in very low abundance (Figures 7A,B). Three main types of *Trichodesmium* spp. were observed during the survey time: *Trichodesmium erythraeum*, *Trichodesmium thiebauti*, and *Trichodesmium hildebrandtii*. The average number of cells per trichome in all samples ranged from 70 to 150 cells/trichome. *Richelia intracellularis is* only detected at station B16 (<30 m) and only formed symbiont with *Rhizomatous rhizophyta* (Figure 7B). The other four types of diazotrophic phylotypes were measured by qPCR. UCYN-A and UCYN-B were generally concentrated in the upper water layers (<75 m). These two types of unicellular cyanobacteria were typically high at Sta. B16 and B20 (Figures 7C,D). γ-HM210363 was the most dominant diazotroph as observed in high-throughput sequencing data. Similarly, qPCR also demonstrated a high abundance of γ-HM210363 in the BoB during the winter monsoon. The gene abundance of γ-HM210363 (copies/L) was two orders of magnitude higher than *Sagittula castanea*, which was recognized as the most abundant species during the pre-southwest monsoon.

### Influence of environmental factors on 16S rRNA gene and *nif*H gene communities

**FIGURE 8:**
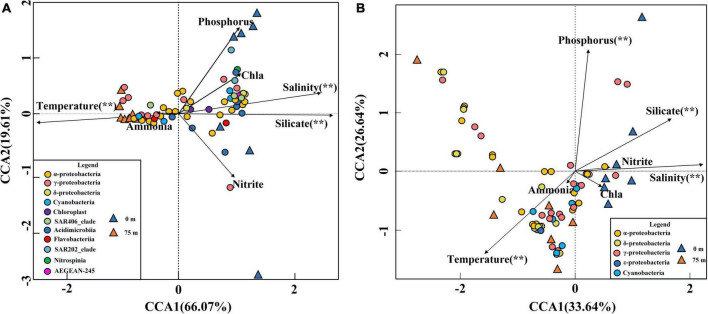
CCA for the relationship between the 16S rRNA gene **(A)** and *nif*H gene **(B)** distributions with the environmental parameters in the BoB. Black arrows represented environmental parameters and different colored triangles represented different layer samples. Significant (**) was determined by 999 Monte Carlo permutation tests in R V.3.0 software.

To assess the influence of environmental factors on the bacterioplankton communities in the BoB, a multivariate analysis (CCA) was performed. In the bacterial community CCA biplot, the first two axes of CCA explained 66.07 and 19.61% of the total variation in the bacterial communities, respectively (Figure 8A). Significant tests of bacterial communities and the environmental factors indicted that temperature, salinity, and silicate contributed significantly (*P* < 0.01) to the total variance, and were closely associated with the first and second axes (999 times Monte–Carlo permutations). Due to the high collinearity with silicate, nitrate was removed from the CCA analysis. The bacterial communities in the CCA biplot also showed a distinct vertical distribution pattern (Figure 8A) similar to NMDS analysis (Figure 3A). The surface samples at Sta. B03, B04, and B12 were positive related to silicate, while in other stations they were more closely related to phosphorus. However, in 75 m samples, the bacterial community composition was mainly influenced by temperature. In addition, the CCA biplot showed that the abundance of most Cyanobacteria, α-Proteobacteria, and γ-Proteobacteria species were positively correlated with temperature, while negatively influenced by nutrients and salinity.

For diazotroph communities, the environmental factors in the first two axes explained 60.28% of the total variance. Significant tests revealed that temperature, phosphorus, salinity, and silicate contributed significantly (*P* < 0.01) to the total variance and were closely associated with the first two axes (999 times Monte–Carlo permutations). In the CCA biplot, surface samples were more closely related to nutrients and salinity, while the 75 m samples were always influenced by temperature (Figure 8B). The relationship between the environmental factors and samples in the *nif*H Gene biplot closely resembled the 16S rRNA gene CCA biplot. The CCA biplot showed that all the Cyanobacteria were positively correlated with temperature while negatively correlated with nutrients and salinity. In addition, we observed that the most abundant three γ-Proteobacteria (OTU1, OTU3, and OTU5) were positively influenced by nutrients and salinity and negatively correlated with temperature. However, the other less abundance γ-Proteobacteria species exhibited the opposite relationship with environmental factors. α-Proteobacteria and β-Proteobacteria also showed the same relationship with environmental factors and most of these species were positively correlated with temperature except for OTU9, OTU30, OTU39, and OTU45.

## Discussion

### Distribution of bacterial communities in the Bay of Bengal during the winter monsoon

Here, we assessed the bacterial communities along a transect across the central BoB with Illumina Hiseq sequencing targeting the V3–V4 region of the 16S rRNA gene. Overall, highly diversified groups of bacterial communities were detected in the study area. Five dominant phyla were reported through the 16S rRNA gene sequencing, among which Proteobacteria and Cyanobacteria were the most abundant phyla than Actinobacteria, Bacteroidetes, and Marinimicrobia. A similar bacterioplankton community structure was also reported earlier in many of the global oligotrophic oceans such as the South China Sea, the Northwestern Pacific Ocean, and the South Atlantic Ocean (Milici et al., 2016; Li et al., 2018; Fernandes et al., 2020). Our results were nevertheless different from recent studies conducted in the coastal areas of BoB (Rajpathak et al., 2018) and the equatorial EIO (Wang et al., 2016) where Cyanobacteria were rarely discovered. This could suggest the distinct spatio-temporal variations within the bacterioplankton communities in the EIO.

The 16S rRNA gene sequencing revealed the distinct distribution pattern within the dominant bacterioplankton groups, i.e., Cyanobacteria, Proteobacteria, Actinobacteria, Chloroflexi, and Marinimicrobia (Supplementary Figure 2). The dominance of the pico-cyanobacteria genus, *Prochlorococcus*, was more abundant in the surface water than in the deeper layers (Figure 3B) and could be attributed to the light-limited conditions in the deeper layers (Rusch et al., 2010). It agreed well with previous reports that *Prochlorococcus* was more abundant at or near the surface, and decreased dramatically along the water column (Beatriz et al., 2016; Kent et al., 2016). The previous study conducted in the neighboring equatorial EIO reported that the mean cell diameter of *Prochlorococcus* was 0.60 ± 0.22 μm, and it further increased with depth (Wei et al., 2018). The smaller *Prochlorococcus* cell on the surface has a competitive advantage over larger phytoplankton for resource acquisition, utilization, and reproduction (Raven, 1998). However, in the deeper layers, their cell diameters are gradually increased to acquire more pigments for growth to compensate for the decreased light levels (Goericke and Welschmeyer, 1998). The *Prochlorococcus* population was less diverse (4 OTUs) in the present study, compared to previous reports where specific primers were used (Kent et al., 2016). *Prochlorococcus* group members shared >97% similarity of their 16S rRNA genes, and this group was always designated as a single species (Babić et al., 2017). Therefore, further investigations targeting the functional gene of *Prochlorococcus* are greatly needed to better understand the real composition of this pico-cyanobacteria in the BoB. *Synechococcus* was another well-recovered Cyanobacteria in this study, though presented in lower relative abundance than *Prochlorococcus*. This group has a wider geographical distribution even in colder, eutrophic waters and high-latitude oceans where *Prochlorococcus* occurs in low abundance or absent (Xia et al., 2019). A recent metagenomic study conducted in the Indian Ocean confirmed the presence of nitrate utilization genes (*nar*B) in *Synechococcus*, which might explain their tolerance to nutrient-rich waters (Beatriz et al., 2016). Like *Prochlorococcus*, 16S rRNA gene sequencing also underestimated the diversity of *Synechococcus* groups (2 OTUs). Two distinct types of *Synechococcus* were observed in the BoB with OTU 4 abundant in the surface waters and OTU 1038 dominant in the 75 m layer. Here possibly OTU 4 group contain higher phycourobilin (bright type) which is always dominant in oligotrophic waters, whereas OTU 1038 was a lower phycourobilin (dim type) containing group which is always found in eutrophic waters (Mazard et al., 2011; Xia et al., 2016). The representative sequence of OTU 338 shared 100% similar with *Richelia* sp. Consistent with *nif*H gene sequencing, the 16S rRNA sequences also revealed a low relative abundance of *Richelia* sp. at all stations. Other nitrogen-fixing cyanobacteria were not detected in the 16S rRNA gene libraries though they might be sequenced.

Compared to Cyanobacteria, Proteobacteria represented the highest diversity and largest fractions in the BoB during the winter monsoon. Among which, α- and γ-Proteobacteria were the most abundant in both two layers, and δ-Proteobacteria was also commonly detected in low abundance. Despite the difference in other dominant species, the dominance of α- and γ-Proteobacteria within the Proteobacteria group report in the BoB agreed with an earlier study from the equatorial EIO (Wang et al., 2016). In α-Proteobacteria, members of the *SAR11* clade and *Rhodobacterales* spp. were most abundant in this study. Here, we have identified four clades of *SAR11* in the BoB according to the previous definition (Supplementary Figure 3). Earlier studies had also reported the α-Proteobacteria clade *SAR11* as the most abundant and diverse group in the global ocean ecosystem (Morris et al., 2002; Rappe et al., 2002). The dominance of this clade in oxygen-rich surface waters as well as in OMZs suggests its adaptive capabilities to confront oxygen decrease (Hawley et al., 2014). The growth of *SAR11* clade also requires exogenous sources of reduced sulfur (e.g., 3-dimethylsulphoniopropionate), suggesting that this group of organisms may be active in sulfur cycles (Morris et al., 2002; Grote et al., 2012). In addition, the efficient genome streamlining of the *SAR11* clade can minimize the cellular nutrient requirements, thus supporting fast growth and replication under extreme oligotrophic conditions (West et al., 2016). The dominant other α-Proteobacteria, *Rhodobacterales* spp., can be correlated with fundamental metabolic processes such as sulfur oxidation, nitrogen fixation, and autotrophic carbon fixation (Lee et al., 2013). Strikingly, the nitrogen fixer *Sagittula castanea* quantified by qPCR in this study belonged to *Rhodobacterales* spp., whereas the phylogenetic analysis revealed that *Sagittula*-related groups were more correlated with the AEGEAN-169 marine group. The relative abundance of another dominant Proteobacteria, γ-Proteobacteria, decreased with depth, which was consistent with the previous study conducted in the North Atlantic Ocean (HéLèNE et al., 2011). Sequence alignment revealed that *Oceanospirillales* and *SAR86* clade were the dominant groups at the order level within γ-Proteobacteria. Some *Oceanospirillales* members were reported to contain genes for carbon fixation (RuBisCO genes) and sulfur oxidation (Swan et al., 2011). In addition, many studies revealed that *Oceanospirillales* members always formed symbiotic with various marine invertebrates (Cao et al., 2014). *SAR86* clade was more abundant in the surface waters. The metagenomic analysis demonstrated that the *SAR86* clade lacks several pathways, such as amino acid and vitamin synthesis as well as sulfate reduction which was commonly detected in other abundant marine microorganisms (Dupont et al., 2012). The genome streamlining strategy supported this group to have a selective advantage over other oligotrophic groups. In δ-Proteobacteria, members of the order *SAR324* clade were abundant in the study area (Supplementary Figure 3). Earlier this clade was reported as a deeper water clade (HéLèNE et al., 2011); however, it has been also detected in the surface waters in our study. This suggests their survival capability in diverse environmental conditions. In brief, adaptive mechanisms such as small cell size and genome streamlining could support the dominance of Proteobacteria in oligotrophic waters of the BoB.

Co-occurrence network analysis revealed that all keystone species belonged to rare species (<0.1% of the total sequences) as also reported in other aquatic ecosystems (Milici et al., 2016; Jia et al., 2018). These “rare biospheres” played a vital role in the process of community assemblage and contributed significantly to the biogeochemical process albeit they only account for a minor fraction of the whole communities (Jiao et al., 2016). In the present study, temperature and salinity were major factors to determine the distribution of bacterial communities. This agreed well with previous studies in many other oceans such as the South China Sea and the Bohai Sea (Zhang et al., 2011; Wu et al., 2022a). Notably, silicate was also the major factor to influence the distribution of bacterial communities in our study. This is because pico-cyanobacteria were the major component of bacterial communities in the BoB. In recent years, pico-cyanobacteria were found to have significant silica accumulation (Wei et al., 2018). Furthermore, nitrate was deleted from the CCA analysis due to the high collinearity with other environments. However, it does not mean that nitrate was not important in structuring the spatial heterogeneity of bacterial communities. It is generally believed that these inorganic nutrient concentrations have important roles in regulating the abundance of various bacterial communities (Wu et al., 2019). According to the PICRUSt simulations, the BoB was losing fixed nitrogen in the deeper layer *via* denitrification and anammox (Figure 6). A decreasing trend of denitrification and anammox was observed from north to south in 75 m layers of the BoB. This suggested that the deeper layer of the BoB was losing a great deal of fixed nitrogen. Thus, we can speculate that the part of the present sampling stations (Sta. B03-B13) was occupied in the OMZs of the BoB. Our results coincided with previous reports by the isotope labeling method which also showed nitrogen loss was significant in OMZs of the BoB (Bristow et al., 2016). However, denitrification and anammox were all suppressed in the surface waters, and the net results were that nitrogen fixation supplied fixed nitrogen in the upper layers. Our previous study using an isotope ^15^N tracer technique also showed that nitrogen fixation rates were high in surface waters in the BoB and the southeastern Indian Ocean (Wu et al., 2022b). Overall, our study demonstrated that nitrogen fixation is significantly important in adding new nitrogen to the upper layers of this oligotrophic marine ecosystem.

### Distribution of diazotroph communities in the Bay of Bengal during the winter monsoon

In the present study, lower diversity of the diazotrophic community was observed in the BoB during the winter monsoon. The diazotrophic communities were present with higher diversity in the surface than deeper layers in contrast to the 16S rRNA gene sequencing. The high diversity of diazotrophic communities on the surface revealed that most of the diazotrophs were lucipetal and more active in the upper ocean. In the phylogenetic analysis, the top 59 OTUs were grouped into two defined clusters of *nif*H genes (Cluster I and Cluster III). Further, these communities were strikingly similar to a recent study conducted in the OMZs of the BoB, so these diazotrophs could be the characteristic of OMZs (Löscher et al., 2020).

Here, the results generated from high-throughput sequencing and microscopic enumeration indicated that nitrogen-fixing Cyanobacteria were not abundant in the study area during the winter monsoon. This was quite different from our earlier study conducted during the spring inter-monsoon, where *Trichodesmium* spp. were dominant in the surface waters of the BoB (Wu et al., 2019, 2022b). Wind-drift current was thought to be the main factor that influences the seasonal rhythm of *Trichodesmium* spp. in the BoB (Hegde et al., 2008; Jyothibabu et al., 2017). *Trichodesmium* colonies can be easily destroyed by strong winds (Capone et al., 1997) and, therefore, the high turbulence during the winter monsoon is not in favor of the growth of *Trichodesmium*. The main types of *Trichodesmium* spp. in the present study were *Trichodesmium thiebauti* and *Trichodesmium erythraeum*. These two species were also reported to appear in both Indian coastal waters and open oceans (Krishnan et al., 2007). Unfortunately, *Trichodesmium* spp. (OTU347) was only retrieved from the *nif*H gene sequencing, while they were not recognized in the 16S rRNA gene sequencing. Unlike *Trichodesmium* spp., *Richelia* spp. was recognized in both gene sequencing though all presented in low abundance. Our results showed that *Richelia* spp. only formed symbionts with *Rhizomatous rhizophyta*, which was different from previous studies conducted in Indian oceans (Kulkarni et al., 2010; Lyimo, 2011).

Except for filamentous Cyanobacteria, unicellular diazotrophs were also detected in both high-throughput sequencing and qPCR in this study. However, the unicellular diazotrophs, including UCYN-A, *Crocosphaera watsonii*, and *Cyanothece* sp. WH 8904, were presented to very low abundance, consistent with our previous study (Wu et al., 2019). The low abundance of unicellular diazotrophs could be attributed to the shallower nitracline depths and continuous high temperature as described earlier (Shiozaki et al., 2014; Wu et al., 2019). Overall, the Cyanobacterial population was not abundant in the central BoB during the winter monsoon. From the neighbor-joining phylogenetic tree, OTU7 shared 100% similarity with UCYN-A3 (ADO20628.1) and was the only species that belonged to UCYN-A among the top 59 OTUs. UCYN-A3 is a newly characterized open ocean sublineage that formed symbiotic with uncultivated unicellular alga (Cornejo-Castillo et al., 2019). Turk-Kubo et al. (2016) reported that UCYN-A3 is always commonly detected in oligotrophic waters and co-occurs with UCYN-A1. However, in this study, UCYN-A1 was not identified in the top 59 OTUs and could be a minor group to detect in this region. Here, UCYN-A was also quantified by qPCR with Taqman assays, and the gene abundance was the pool of UCYN-A2, UCYN-A3, and UCYN-A4 as stated elsewhere (Henke et al., 2018). Similar to high-throughput sequencing, qPCR also showed UCYN-A sublineages presented in low abundance toward the surface layers.

Unlike the Cyanobacteria, Proteobacteria represented the highest fractions of diazotrophs in the BoB during the winter monsoon. Here, the diazotroph communities mainly consisted of heterotrophic γ-Proteobacteria, which was significantly different from our previous pre-southwest monsoon study, where α-Proteobacteria dominated at the class level (Wu et al., 2019). The sequencing and qPCR analysis demonstrated that OTU1 (γ-HM210363) was the most abundant species and omnipresent in the BoB as compared with other proteobacterial subgroups. γ-HM210363 was once reported as relatively ubiquitous in the South Pacific Gyre, whereas not necessarily in the gene numbers (Halm et al., 2012). The gene abundance of *Sagittula castanea* was one or two orders of magnitude lower than that during the pre-southwest monsoon (Wu et al., 2019), which indicated high turbulence suppressing the growth of *Sagittula castanea*. Proteobacteria also exhibited significant temporal differences, where Cluster III was commonly detected in this study. Cluster III groups consisted mostly of sulfate-reducing bacteria and were always dominant in oxygen-depleted waters (Cheung et al., 2015). The subsurface waters in the BoB contain extremely low, but persistent concentrations of oxygen in the nanomolar range (Bristow et al., 2016). Thus, the low oxygen concentrations favor the growth of Cluster III species. However, here strangely Cluster III species were most detected in the surface waters, while it was sporadically detected in the 75 m layers. This discrepancy could be caused due to PCR bias because γ-Proteobacteria accounted for the majority of the diazotroph communities in the 75 m layers.

To date, none of the studies are focused on the assembly process of marine diazotroph communities in the oceans. Our study demonstrated that diazotroph communities were highly modular, and Proteobacteria played a vital role in the process of community assembling (Figures 5C,D). Strikingly, all recognized keystone species were rare species (<0.1% of the total sequences) and identical to the bacterial network. In this respect, the “rare biosphere” mediated multiple metabolic capabilities and ecosystem functions including nitrogen cycles (Jia et al., 2018). The CCA biplot of diazotroph communities explained less total variance (60.28%) in the present study than that during the pre-southwest monsoon (83.6%) (Wu et al., 2019). Wind stress was presumably to be a neglected parameter that could explain a considerable variance in the present study. The rest significant environmental parameters (temperature, salinity, phosphorus, and silicate) in the CCA biplot were generally the same as our study conducted during pre-southwest monsoon after excluding highly collinearity parameters (Wu et al., 2019). This might be caused by different diazotroph communities that are suitable for living in different gradients of temperature and salinity. For example, UCYN-A tended to live in high salinity but low-temperature seawater, whereas *Trichodesmium* spp. tended to thrive when the temperature is 25°C or warmer (Wu et al., 2018; Li et al., 2021; Christian et al., 2022). Phosphorus is also an important factor to determine the distribution of diazotroph communities in our study. This might be the cause that phosphorus is the major component of cells and is thought to be a key factor in limiting diazotroph communities and their nitrogen fixation (Wu et al., 2019). Notably, we observed that the influence of silicate on diazotroph communities is also significant in our study. This phenomenon was also detected by a recent study conducted in the Eastern Indian Ocean and might be attributed to some heterotrophic diazotrophs entering a mutualistic symbiosis with diatoms (Wu et al., 2022b).

## Conclusion

Here, our research focused on how the diazotroph communities play a significant role in the bacterioplankton communities, and their genetic potential to carry out nitrogen fixation. Generally, this study provided a comprehensive and intensive investigation of bacterial and diazotroph communities in the open oceans of the BoB during the winter monsoon. Although a recent study has analyzed the diazotroph communities based on clone library in the same region, our study provided a deeper sequencing of diazotroph communities as well as the bacterial communities based on high-throughput sequencing. The sequencing analysis presented that 75 m layers had higher diversity than surface samples of the bacterial communities, while diazotroph communities were presented with a reverse trend. Proteobacteria were the most abundant groups at the phylum level in both bacterial and diazotroph communities. Combined with our previous study, we found that both bacterial and diazotroph communities exhibited distinct spatio-temporal variations in the BoB. Co-occurrence networks demonstrated that rare biosphere might play a significant role in the assemble process of both bacterial and diazotroph communities. Limited by sequencing depth, our study lost some extremely rare OTUs and that is why we did not conduct a more in-depth analysis of abundant and rare communities. Further studies based on deeper sequencing are greatly in need to better understand the coupling and response between abundant and rare populations.

## Data availability statement

The 16S rRNA gene and *nif*H gene raw sequences obtained from this study were deposited in NCBI Sequence Read Archive with BioProject nos. PRJNA637963 and PRJNA637983, respectively.

## Author contributions

CW: investigation, methodology, software, validation, formal analysis, data curation, visualization, writing – original draft, and writing – review and editing. DN: software, writing – review and editing, and supervision. ZC: writing – review and editing and supervision. XW, WX, and GZ: investigation, writing – review and editing, and supervision. JS: investigation, writing – review and editing, supervision, project administration, and funding acquisition. All authors contributed to the article and approved the submitted version.

## Footnotes

1
http://www.biocloud.net/

2
https://chao.shinyapps.io/iNEXTOnline/
