# Supplementary material for: Diversity, structure, and distribution of bacterioplankton and diazotroph communities in the Bay of Bengal during the winter monsoon
Source: Front Microbiol. 2022 Nov 30;13:987462. doi: 10.3389/fmicb.2022.987462 (PMC9748438; doi:10.3389/fmicb.2022.987462)
Supplement: Supplementary file 1 [file Data_Sheet_1.docx]

**Diversity, structure, and distribution of** **bacterioplankton and diazotroph communities in the Bay of Bengal during the winter monsoon**

Chao Wu ^1,2^, DHIRAJ Dhondiram Narale^3^, Zhengguo Cui ^1,2^, Xingzhou Wang ^3^, Haijiao Liu ^3^, Wenzhe Xu ^3^, Guicheng Zhang ^3^, Jun Sun ^3,4*^

^1^ Key Laboratory of Sustainable Development of Marine Fisheries, Ministry of Agriculture and Rural Affairs, Yellow Sea Fisheries Research Institute, Chinese Academy of Fishery Sciences, Qingdao, China

^2^ Laboratory for Marine Fisheries Science and Food Production Processes, Pilot National Laboratory for Marine Science and Technology, Qingdao, China

^3^ Research Centre for Indian Ocean Ecosystem, Tianjin University of Science and Technology, Tianjin, China
^4^ College of Marine Science and Technology, China University of Geosciences (Wuhan), Wuhan, China

*Corresponding author

e-mail: [phytoplankton@163.com](mailto:phytoplankton@163.com)

**
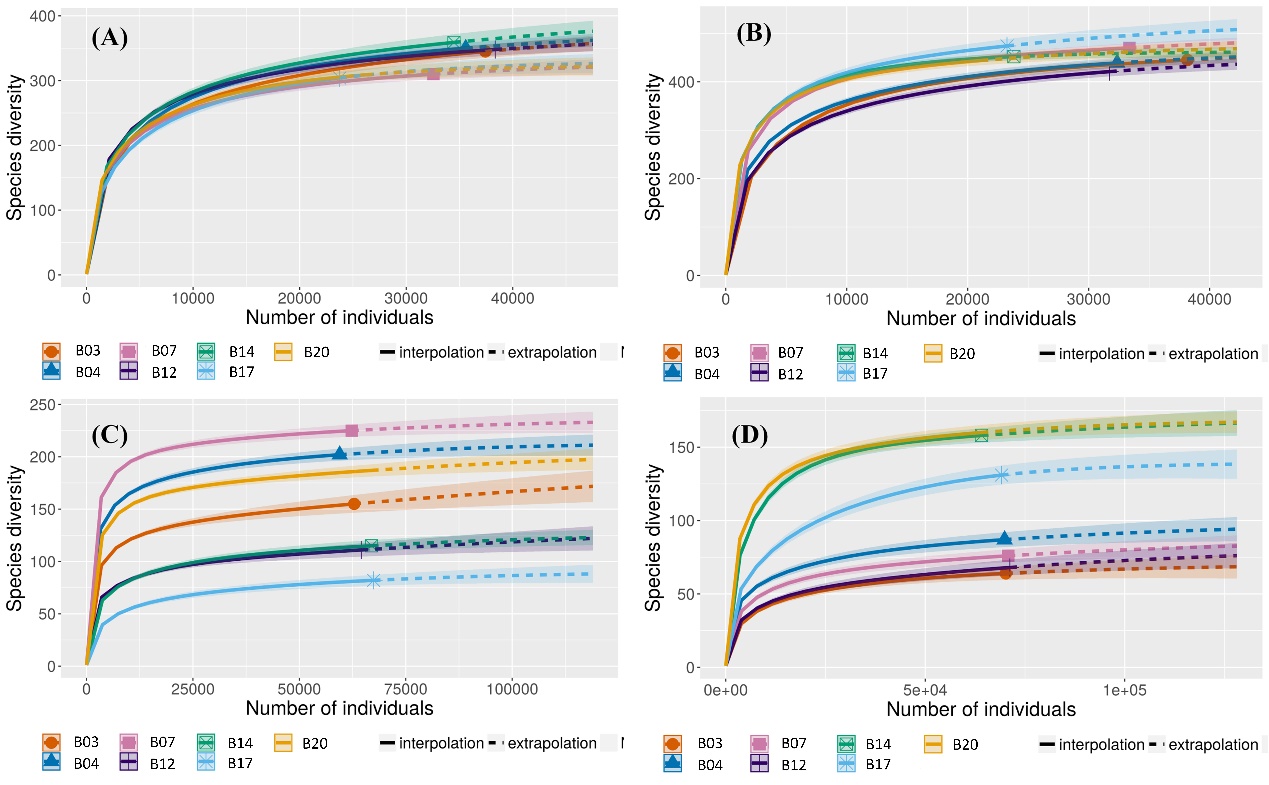
**

**Supplemental Figure S1** **Rarefaction curves of** **16S rRNA gene (A:0 m; B: 75 m) and *nif*H gene (C:0 m; D: 75 m) which compares the number of effective sequences with the number of** **phylotypes (OTUs).**

**
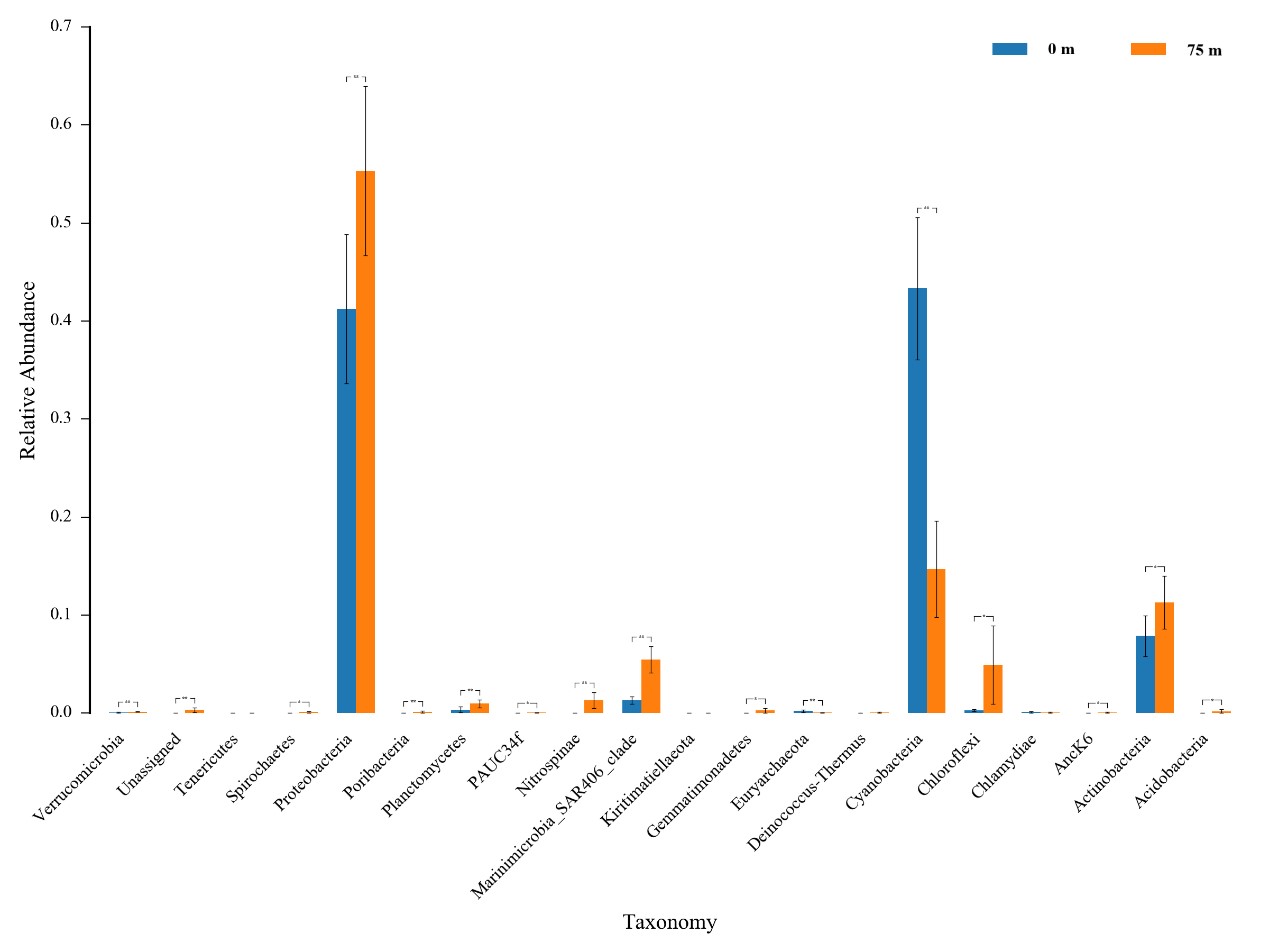
**

**Supplemental Figure S2 Histogram of ANOVA between groups at the kingdom level. Note that X-axis represents species (The top 20 species with the lowest p values are shown); Y-axis represents the relative richness of species; Columns with different colours represent samples, * on the columns means that the difference is significant (p<0.05) while ** means the difference is very significant (p<0.01).**

**
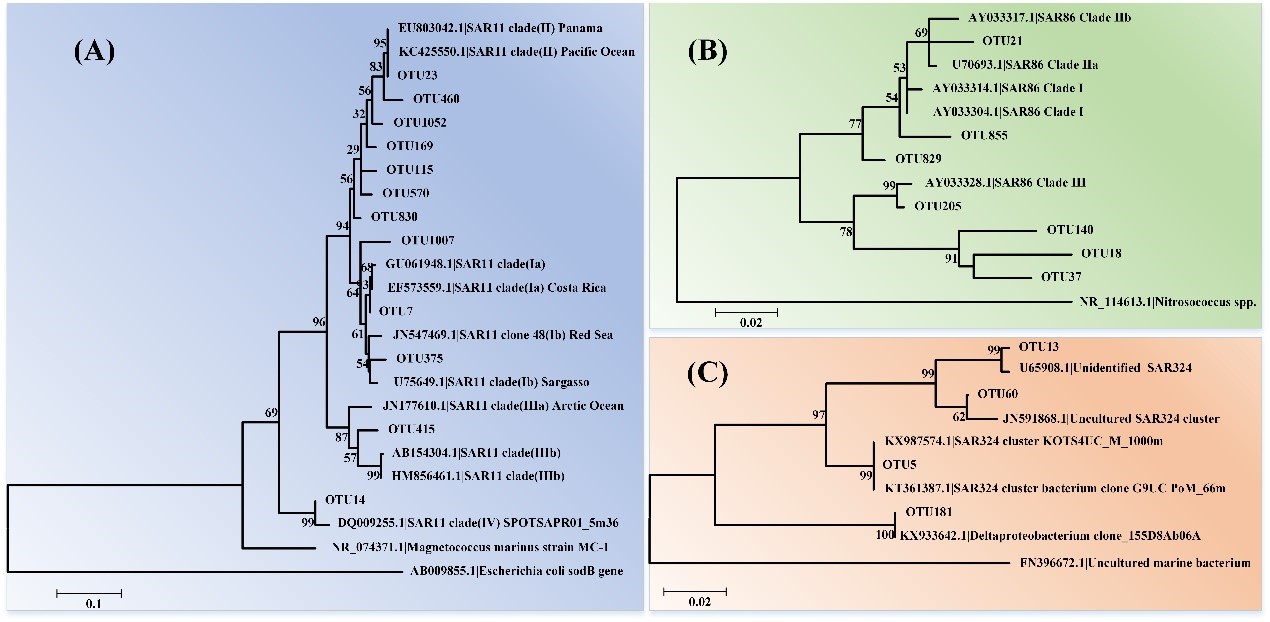
**

**Supplemental Figure S3 Expanded clusters of the grouped sequences of SAR11 (A), SAR 86 (B) and SAR 324 (C). The scale bar represents 10% estimated sequence divergence**

**Supplemental Table S1 Temperature (T), salinity (S), chlorophyll a (Chl a) concentration, and dissolved inorganic nutrients (ammonium, nitrate, nitrite, phosphate) insurface water at sampling stations**

| Stations | Temperature  (°C) | Salinity | Phosphorus  (μM) | Ammonia  (μM) | Nitrate  (μM) | Nitrite  (μM) | Silicate  (μM) | Chla  (μg L^-1^) |
| --- | --- | --- | --- | --- | --- | --- | --- | --- |
| B03-0m | 28.53 | 33.12 | 0.57 | 1.57 | 0.30 | 0.09 | 0.98 | 0.29 |
| B03-75m | 27.26 | 34.34 | 0.00 | 1.57 | 7.70 | 0.16 | 2.88 | 0.30 |
| B04-0m | 28.57 | 33.17 | 0.33 | 1.07 | 0.26 | 0.04 | 0.87 | 0.28 |
| B04-75m | 26.87 | 34.22 | 0.39 | 0.19 | 7.62 | 0.37 | 4.79 | 0.57 |
| B07-0m | 28.79 | 33.77 | 0.05 | 0.94 | 0.46 | 0.06 | 0.94 | 0.09 |
| B07-75m | 25.45 | 34.72 | 0.58 | 1.31 | 6.49 | 0.12 | 3.65 | 0.12 |
| B12-0m | 28.93 | 34.04 | 0.05 | 1.53 | 1.56 | 0.14 | 1.26 | 0.39 |
| B12-75m | 23.61 | 34.99 | 0.60 | 0.70 | 9.20 | 0.05 | 5.08 | 0.18 |
| B13-0m | 28.50 | 34.19 | 0.02 | 2.64 | 2.21 | 0.16 | 0.88 | 0.12 |
| B13-75m | 25.14 | 34.92 | 0.38 | 0.44 | 6.90 | 0.05 | 4.22 | 1.15 |
| B16-0m | 28.84 | 33.91 | 0.03 | 1.17 | 0.78 | 0.06 | 0.56 | 0.11 |
| B16-75m | 27.46 | 34.87 | 0.20 | 1.07 | 2.48 | 0.07 | 1.55 | 0.53 |
| B20-0m | 29.30 | 34.26 | 0.06 | 0.63 | 0.56 | 0.02 | 0.79 | 0.22 |
| B20-75m | 28.21 | 34.74 | 0.37 | 3.12 | 7.07 | 0.11 | 3.89 | 0.25 |

**Supplemental Table S2 Diversity and predicted richness of *nif*H sequences recovered from the EIO by Illumina Hiseq2500 platform. Note that *nif*H gene diversity was calculated after removing OTUs contained less than 10 sequences across all samples.**

| Sites | | B03 | B04 | B07 | B12 | B13 | B16 | B20 |
| --- | --- | --- | --- | --- | --- | --- | --- | --- |
| 0m (16S rRNA) | Effective Tags | 37188 | 35405 | 32379 | 38070 | 34249 | 23653 | 26023 |
|  | No. of OTUs | 339 | 344 | 304 | 342 | 353 | 299 | 303 |
|  | Coverage (C%) | 0.9985 | 0.9987 | 0.999 | 0.9989 | 0.9984 | 0.998 | 0.9987 |
|  | Chao1 | 379.1351 | 370.5385 | 321.1034 | 366.375 | 401.125 | 324.7381 | 317.6667 |
|  | Shannon-Weiner | 3.2449 | 2.9065 | 3.416 | 3.4146 | 3.2964 | 2.8145 | 3.1741 |
|  | Simpson | 0.1271 | 0.2248 | 0.1193 | 0.1023 | 0.1114 | 0.2238 | 0.1568 |
| 75m (16S rRNA) | Effective Tags | 38027 | 32161 | 33140 | 31538 | 23638 | 23085 | 21020 |
|  | No. of OTUs | 445 | 435 | 467 | 417 | 450 | 470 | 442 |
|  | Coverage (C%) | 0.9987 | 0.9984 | 0.9987 | 0.9982 | 0.9989 | 0.9973 | 0.9979 |
|  | Chao1 | 476.7838 | 465.3571 | 501.7308 | 449.0833 | 459.75 | 531.0312 | 475.7857 |
|  | Shannon-Weiner | 3.6158 | 4.2535 | 4.667 | 4.074 | 4.7273 | 4.7416 | 4.7222 |
|  | Simpson | 0.1023 | 0.0329 | 0.0229 | 0.0409 | 0.0208 | 0.0189 | 0.0198 |
| 0m (*nif*H gene) | Effective Tags | 60886 | 58422 | 60647 | 62919 | 65381 | 66732 | 65945 |
|  | No. of OTUs | 106 | 155 | 181 | 67 | 65 | 44 | 142 |
|  | Coverage (C%) | 99.83 | 99.73 | 99.70 | 99.89 | 99.9 | 99.93 | 99.78 |
|  | Chao1 | 172 | 163.3 | 188.9 | 89.5 | 67.5 | 47.5 | 164.8 |
|  | Shannon-Weiner | 2.7151 | 2.3497 | 3.5252 | 2.4704 | 1.7824 | 1.1218 | 2.5755 |
|  | Simpson | 0.8969 | 0.6978 | 0.9431 | 0.8718 | 0.6589 | 0.5851 | 0.7910 |
| 75m (*nif*H gene) | Effective Tags | 67492 | 67137 | 67720 | 68175 | 61548 | 66724 | 61875 |
|  | No. of OTUs | 14 | 33 | 24 | 16 | 100 | 75 | 107 |
|  | Coverage (C%) | 99.98 | 99.95 | 99.96 | 99.98 | 99.85 | 99.89 | 99.83 |
|  | Chao1 | 24.5 | 40.5 | 27 | 21 | 105.3 | 77.5 | 109 |
|  | Shannon-Weiner | 0.2304 | 0.8339 | 1.0262 | 0.8791 | 1.3559 | 1.2250 | 1.4718 |
|  | Simpson | 0.1057 | 0.4446 | 0.5727 | 0.5092 | 0.5799 | 0.5297 | 0.6441 |
